# Supplementary material for: Time‑resolved multi-omic analysis of paclitaxel exposure in human iPSC‑derived sensory neurons unveils mechanisms of chemotherapy‑induced peripheral neuropathy
Source: Cell Death Dis. 2026 Feb 10;17(1):211. doi: 10.1038/s41419-026-08445-2 (PMC12921266; doi:10.1038/s41419-026-08445-2)
Supplement: Supplementary file 15 — Dataset 1. [file 41419_2026_8445_MOESM15_ESM.zip › CDD_Open_Data/04_RNA_Seq/Timeline_panel_plots_etc_2023-01-23_1609.zip!/Panel_Plots_etc/Statistics_Summary.docx]

| **Field** | **Value** |
| --- | --- |
| Gene set ID | M5924 |
| Description | HALLMARK_MTORC1_SIGNALING |
| Data set | standard contrasts |
| Contrast | 72h_PTX_vs_DMSO_ID0 |
| Database | msigdb_hallmark |
| Sort order | pval |
| P value | <2e-16 |
| adj. P value | <2e-16 |
| Effect size (AUC) | 0.67 |
| Field | Value |
| Gene set ID | M5930 |
| Description | HALLMARK_EPITHELIAL_MESENCHYMAL_TRANSITION |
| Data set | standard contrasts |
| Contrast | 72h_PTX_vs_DMSO_ID0 |
| Database | msigdb_hallmark |
| Sort order | pval |
| P value | <2e-16 |
| adj. P value | 2.4e-16 |
| Effect size (AUC) | 0.64 |
| Field | Value |
| Gene set ID | M5908 |
| Description | HALLMARK_ANDROGEN_RESPONSE |
| Data set | standard contrasts |
| Contrast | 72h_PTX_vs_DMSO_ID0 |
| Database | msigdb_hallmark |
| Sort order | pval |
| P value | 2.9e-12 |
| adj. P value | 2.9e-11 |
| Effect size (AUC) | 0.63 |
| Field | Value |
| Gene set ID | M5890 |
| Description | HALLMARK_TNFA_SIGNALING_VIA_NFKB |
| Data set | standard contrasts |
| Contrast | 24h_PTX_vs_DMSO_ID4 |
| Database | msigdb_hallmark |
| Sort order | pval |
| P value | <2e-16 |
| adj. P value | <2e-16 |
| Effect size (AUC) | 0.65 |
| Field | Value |
| Gene set ID | M5908 |
| Description | HALLMARK_ANDROGEN_RESPONSE |
| Data set | standard contrasts |
| Contrast | 72h_PTX_vs_DMSO_ID0 |
| Database | msigdb_hallmark |
| Sort order | pval |
| P value | 2.9e-12 |
| adj. P value | 2.9e-11 |
| Effect size (AUC) | 0.63 |
| Field | Value |
| Gene set ID | M5944 |
| Description | HALLMARK_ANGIOGENESIS |
| Data set | standard contrasts |
| Contrast | 72h_PTX_vs_DMSO_ID0 |
| Database | msigdb_hallmark |
| Sort order | pval |
| P value | 9.5e-05 |
| adj. P value | 0.00025 |
| Effect size (AUC) | 0.68 |
| Field | Value |
| Gene set ID | M5890 |
| Description | HALLMARK_TNFA_SIGNALING_VIA_NFKB |
| Data set | standard contrasts |
| Contrast | 24h_PTX_vs_DMSO_ID4 |
| Database | msigdb_hallmark |
| Sort order | pval |
| P value | <2e-16 |
| adj. P value | <2e-16 |
| Effect size (AUC) | 0.65 |
| Field | Value |
| Gene set ID | M5924 |
| Description | HALLMARK_MTORC1_SIGNALING |
| Data set | standard contrasts |
| Contrast | 48h_PTX_vs_DMSO_ID5 |
| Database | msigdb_hallmark |
| Sort order | pval |
| P value | <2e-16 |
| adj. P value | <2e-16 |
| Effect size (AUC) | 0.65 |
| Field | Value |
| Gene set ID | M5892 |
| Description | HALLMARK_CHOLESTEROL_HOMEOSTASIS |
| Data set | standard contrasts |
| Contrast | 5d_PTX_vs_DMSO_ID6 |
| Database | msigdb_hallmark |
| Sort order | pval |
| P value | <2e-16 |
| adj. P value | <2e-16 |
| Effect size (AUC) | 0.77 |
| Field | Value |
| Gene set ID | M5890 |
| Description | HALLMARK_TNFA_SIGNALING_VIA_NFKB |
| Data set | standard contrasts |
| Contrast | 5d_PTX_vs_DMSO_ID6 |
| Database | msigdb_hallmark |
| Sort order | pval |
| P value | 1.7e-15 |
| adj. P value | 2.9e-14 |
| Effect size (AUC) | 0.66 |
| Field | Value |
| Gene set ID | LI.M35.0 |
| Description | signaling in T cells (I) |
| Data set | standard contrasts |
| Contrast | 24h_PTX_vs_DMSO_ID4 |
| Database | tmod |
| Sort order | pval |
| P value | 3.8e-06 |
| adj. P value | 0.0023 |
| Effect size (AUC) | 0.67 |
| Field | Value |
| Gene set ID | LI.M20 |
| Description | AP-1 transcription factor network |
| Data set | standard contrasts |
| Contrast | 24h_PTX_vs_DMSO_ID4 |
| Database | tmod |
| Sort order | pval |
| P value | 2.2e-05 |
| adj. P value | 0.0045 |
| Effect size (AUC) | 0.61 |
| Field | Value |
| Gene set ID | LI.M35.0 |
| Description | signaling in T cells (I) |
| Data set | standard contrasts |
| Contrast | 48h_PTX_vs_DMSO_ID5 |
| Database | tmod |
| Sort order | pval |
| P value | 5.2e-07 |
| adj. P value | 0.00032 |
| Effect size (AUC) | 0.77 |
| Field | Value |
| Gene set ID | LI.M20 |
| Description | AP-1 transcription factor network |
| Data set | standard contrasts |
| Contrast | 48h_PTX_vs_DMSO_ID5 |
| Database | tmod |
| Sort order | pval |
| P value | 2.6e-06 |
| adj. P value | 8e-04 |
| Effect size (AUC) | 0.73 |
| Field | Value |
| Gene set ID | LI.M35.0 |
| Description | signaling in T cells (I) |
| Data set | standard contrasts |
| Contrast | 72h_PTX_vs_DMSO_ID0 |
| Database | tmod |
| Sort order | pval |
| P value | 3.5e-06 |
| adj. P value | 0.0015 |
| Effect size (AUC) | 0.72 |
| Field | Value |
| Gene set ID | LI.M2.0 |
| Description | extracellular matrix (I) |
| Data set | standard contrasts |
| Contrast | 72h_PTX_vs_DMSO_ID0 |
| Database | tmod |
| Sort order | pval |
| P value | 2.6e-05 |
| adj. P value | 0.0054 |
| Effect size (AUC) | 0.66 |
| Field | Value |
| Gene set ID | M16227 |
| Description | REACTOME_CHOLESTEROL_BIOSYNTHESIS |
| Data set | standard contrasts |
| Contrast | 24h_PTX_vs_DMSO_ID4 |
| Database | msigdb_reactome |
| Sort order | pval |
| P value | <2e-16 |
| adj. P value | 2.5e-15 |
| Effect size (AUC) | 0.86 |
| Field | Value |
| Gene set ID | M27172 |
| Description | REACTOME_ACTIVATION_OF_GENE_EXPRESSION_BY_SREBF_SREBP |
| Data set | standard contrasts |
| Contrast | 24h_PTX_vs_DMSO_ID4 |
| Database | msigdb_reactome |
| Sort order | pval |
| P value | 3.5e-15 |
| adj. P value | 2.8e-12 |
| Effect size (AUC) | 0.71 |
| Field | Value |
| Gene set ID | M27001 |
| Description | REACTOME_REGULATION_OF_CHOLESTEROL_BIOSYNTHESIS_BY_SREBP_SREBF |
| Data set | standard contrasts |
| Contrast | 24h_PTX_vs_DMSO_ID4 |
| Database | msigdb_reactome |
| Sort order | pval |
| P value | 7.8e-13 |
| adj. P value | 4.2e-10 |
| Effect size (AUC) | 0.62 |
| Field | Value |
| Gene set ID | M27172 |
| Description | REACTOME_ACTIVATION_OF_GENE_EXPRESSION_BY_SREBF_SREBP |
| Data set | standard contrasts |
| Contrast | 24h_PTX_vs_DMSO_ID4 |
| Database | msigdb_reactome |
| Sort order | pval |
| P value | 3.5e-15 |
| adj. P value | 2.8e-12 |
| Effect size (AUC) | 0.71 |
| Gene set ID | M27001 |
| Description | REACTOME_REGULATION_OF_CHOLESTEROL_BIOSYNTHESIS_BY_SREBP_SREBF |
| Data set | standard contrasts |
| Contrast | 24h_PTX_vs_DMSO_ID4 |
| Database | msigdb_reactome |
| Sort order | pval |
| P value | 7.8e-13 |
| adj. P value | 4.2e-10 |
| Effect size (AUC) | 0.62 |
| Field | Value |
| Gene set ID | M16227 |
| Description | REACTOME_CHOLESTEROL_BIOSYNTHESIS |
| Data set | standard contrasts |
| Contrast | 48h_PTX_vs_DMSO_ID5 |
| Database | msigdb_reactome |
| Sort order | pval |
| P value | <2e-16 |
| adj. P value | <2e-16 |
| Effect size (AUC) | 0.91 |
| Field | Value |
| Gene set ID | M27172 |
| Description | REACTOME_ACTIVATION_OF_GENE_EXPRESSION_BY_SREBF_SREBP |
| Data set | standard contrasts |
| Contrast | 48h_PTX_vs_DMSO_ID5 |
| Database | msigdb_reactome |
| Sort order | pval |
| P value | <2e-16 |
| adj. P value | <2e-16 |
| Effect size (AUC) | 0.7 |
| Gene set ID | M27001 |
| Description | REACTOME_REGULATION_OF_CHOLESTEROL_BIOSYNTHESIS_BY_SREBP_SREBF |
| Data set | standard contrasts |
| Contrast | 48h_PTX_vs_DMSO_ID5 |
| Database | msigdb_reactome |
| Sort order | pval |
| P value | <2e-16 |
| adj. P value | <2e-16 |
| Effect size (AUC) | 0.65 |
| Field | Value |
| Gene set ID | M29832 |
| Description | REACTOME_RESPONSE_OF_EIF2AK1_HRI_TO_HEME_DEFICIENCY |
| Data set | standard contrasts |
| Contrast | 48h_PTX_vs_DMSO_ID5 |
| Database | msigdb_reactome |
| Sort order | pval |
| P value | 2.2e-09 |
| adj. P value | 7.2e-07 |
| Effect size (AUC) | 0.8 |
| Gene set ID | M27934 |
| Description | REACTOME_ASSEMBLY_AND_CELL_SURFACE_PRESENTATION_OF_NMDA_RECEPTORS |
| Data set | standard contrasts |
| Contrast | 48h_PTX_vs_DMSO_ID5 |
| Database | msigdb_reactome |
| Sort order | pval |
| P value | 3e-08 |
| adj. P value | 6.8e-06 |
| Effect size (AUC) | 0.69 |

| **Field** | **Value** |
| --- | --- |
| Gene set ID | M16227 |
| Description | REACTOME_CHOLESTEROL_BIOSYNTHESIS |
| Data set | standard contrasts |
| Contrast | 72h_PTX_vs_DMSO_ID0 |
| Database | msigdb_reactome |
| Sort order | pval |
| P value | <2e-16 |
| adj. P value | <2e-16 |
| Effect size (AUC) | 0.87 |
| Field | Value |
| Gene set ID | M27172 |
| Description | REACTOME_ACTIVATION_OF_GENE_EXPRESSION_BY_SREBF_SREBP |
| Data set | standard contrasts |
| Contrast | 72h_PTX_vs_DMSO_ID0 |
| Database | msigdb_reactome |
| Sort order | pval |
| P value | <2e-16 |
| adj. P value | <2e-16 |
| Effect size (AUC) | 0.7 |
| Field | Value |
| Gene set ID | M27832 |
| Description | REACTOME_METABOLISM_OF_STEROIDS |
| Data set | standard contrasts |
| Contrast | 72h_PTX_vs_DMSO_ID0 |
| Database | msigdb_reactome |
| Sort order | pval |
| P value | <2e-16 |
| adj. P value | <2e-16 |
| Effect size (AUC) | 0.62 |
| Field | Value |
| Gene set ID | M27001 |
| Description | REACTOME_REGULATION_OF_CHOLESTEROL_BIOSYNTHESIS_BY_SREBP_SREBF |
| Data set | standard contrasts |
| Contrast | 72h_PTX_vs_DMSO_ID0 |
| Database | msigdb_reactome |
| Sort order | pval |
| P value | <2e-16 |
| adj. P value | <2e-16 |
| Effect size (AUC) | 0.67 |
| Field | Value |
| Gene set ID | M29832 |
| Description | REACTOME_RESPONSE_OF_EIF2AK1_HRI_TO_HEME_DEFICIENCY |
| Data set | standard contrasts |
| Contrast | 72h_PTX_vs_DMSO_ID0 |
| Database | msigdb_reactome |
| Sort order | pval |
| P value | 7.5e-12 |
| adj. P value | 1.7e-09 |
| Effect size (AUC) | 0.89 |
| Field | Value |
| Gene set ID | M26917 |
| Description | REACTOME_RAF_INDEPENDENT_MAPK1_3_ACTIVATION |
| Data set | standard contrasts |
| Contrast | 72h_PTX_vs_DMSO_ID0 |
| Database | msigdb_reactome |
| Sort order | pval |
| P value | 1.1e-06 |
| adj. P value | 0.00011 |
| Effect size (AUC) | 0.7 |
| Field | Value |
| Gene set ID | M16227 |
| Description | REACTOME_CHOLESTEROL_BIOSYNTHESIS |
| Data set | standard contrasts |
| Contrast | 5d_PTX_vs_DMSO_ID6 |
| Database | msigdb_reactome |
| Sort order | pval |
| P value | <2e-16 |
| adj. P value | <2e-16 |
| Effect size (AUC) | 0.87 |
| Field | Value |
| Gene set ID | M27172 |
| Description | REACTOME_ACTIVATION_OF_GENE_EXPRESSION_BY_SREBF_SREBP |
| Data set | standard contrasts |
| Contrast | 5d_PTX_vs_DMSO_ID6 |
| Database | msigdb_reactome |
| Sort order | pval |
| P value | <2e-16 |
| adj. P value | <2e-16 |
| Effect size (AUC) | 0.73 |
| Field | Value |
| Gene set ID | M27001 |
| Description | REACTOME_REGULATION_OF_CHOLESTEROL_BIOSYNTHESIS_BY_SREBP_SREBF |
| Data set | standard contrasts |
| Contrast | 5d_PTX_vs_DMSO_ID6 |
| Database | msigdb_reactome |
| Sort order | pval |
| P value | <2e-16 |
| adj. P value | <2e-16 |
| Effect size (AUC) | 0.68 |
| Field | Value |
| Gene set ID | M27832 |
| Description | REACTOME_METABOLISM_OF_STEROIDS |
| Data set | standard contrasts |
| Contrast | 5d_PTX_vs_DMSO_ID6 |
| Database | msigdb_reactome |
| Sort order | pval |
| P value | <2e-16 |
| adj. P value | <2e-16 |
| Effect size (AUC) | 0.62 |
| Field | Value |
| Gene set ID | M735 |
| Description | REACTOME_NEURONAL_SYSTEM |
| Data set | standard contrasts |
| Contrast | 5d_PTX_vs_DMSO_ID6 |
| Database | msigdb_reactome |
| Sort order | pval |
| P value | <2e-16 |
| adj. P value | <2e-16 |
| Effect size (AUC) | 0.65 |
| Field | Value |
| Gene set ID | M15514 |
| Description | REACTOME_TRANSMISSION_ACROSS_CHEMICAL_SYNAPSES |
| Data set | standard contrasts |
| Contrast | 5d_PTX_vs_DMSO_ID6 |
| Database | msigdb_reactome |
| Sort order | pval |
| P value | <2e-16 |
| adj. P value | 2e-15 |
| Effect size (AUC) | 0.66 |
| Field | Value |
| Gene set ID | M752 |
| Description | REACTOME_NEUROTRANSMITTER_RECEPTORS_AND_POSTSYNAPTIC_SIGNAL_TRANSMISSION |
| Data set | standard contrasts |
| Contrast | 5d_PTX_vs_DMSO_ID6 |
| Database | msigdb_reactome |
| Sort order | pval |
| P value | 6.4e-11 |
| adj. P value | 1.3e-08 |
| Effect size (AUC) | 0.65 |
| Field | Value |
| Gene set ID | M4281 |
| Description | REACTOME_NEUROTRANSMITTER_RELEASE_CYCLE |
| Data set | standard contrasts |
| Contrast | 5d_PTX_vs_DMSO_ID6 |
| Database | msigdb_reactome |
| Sort order | pval |
| P value | 5.2e-08 |
| adj. P value | 7e-06 |
| Effect size (AUC) | 0.69 |
| Field | Value |
| Gene set ID | M927 |
| Description | REACTOME_GABA_SYNTHESIS_RELEASE_REUPTAKE_AND_DEGRADATION |
| Data set | standard contrasts |
| Contrast | 5d_PTX_vs_DMSO_ID6 |
| Database | msigdb_reactome |
| Sort order | pval |
| P value | 1.2e-07 |
| adj. P value | 1.5e-05 |
| Effect size (AUC) | 0.77 |
| Field | Value |
| Gene set ID | M27617 |
| Description | REACTOME_PROTEIN_PROTEIN_INTERACTIONS_AT_SYNAPSES |
| Data set | standard contrasts |
| Contrast | 5d_PTX_vs_DMSO_ID6 |
| Database | msigdb_reactome |
| Sort order | pval |
| P value | 1.8e-06 |
| adj. P value | 0.00015 |
| Effect size (AUC) | 0.67 |
| Field | Value |
| Gene set ID | M27018 |
| Description | REACTOME_NEUROTOXICITY_OF_CLOSTRIDIUM_TOXINS |
| Data set | standard contrasts |
| Contrast | 5d_PTX_vs_DMSO_ID6 |
| Database | msigdb_reactome |
| Sort order | pval |
| P value | 1.8e-06 |
| adj. P value | 0.00015 |
| Effect size (AUC) | 0.79 |
| Field | Value |
| Gene set ID | M26917 |
| Description | REACTOME_RAF_INDEPENDENT_MAPK1_3_ACTIVATION |
| Data set | standard contrasts |
| Contrast | 5d_PTX_vs_DMSO_ID6 |
| Database | msigdb_reactome |
| Sort order | pval |
| P value | 2e-06 |
| adj. P value | 0.00016 |
| Effect size (AUC) | 0.7 |
| Field | Value |
| Gene set ID | M7455 |
| Description | REACTOME_INTRINSIC_PATHWAY_FOR_APOPTOSIS |
| Data set | standard contrasts |
| Contrast | 5d_PTX_vs_DMSO_ID6 |
| Database | msigdb_reactome |
| Sort order | pval |
| P value | 1e-05 |
| adj. P value | 6e-04 |
| Effect size (AUC) | 0.64 |
| Field | Value |
| Gene set ID | M27949 |
| Description | REACTOME_LONG_TERM_POTENTIATION |
| Data set | standard contrasts |
| Contrast | 5d_PTX_vs_DMSO_ID6 |
| Database | msigdb_reactome |
| Sort order | pval |
| P value | 2.8e-05 |
| adj. P value | 0.0013 |
| Effect size (AUC) | 0.8 |
| Field | Value |
| Gene set ID | M7223 |
| Description | REACTOME_TRAFFICKING_OF_AMPA_RECEPTORS |
| Data set | standard contrasts |
| Contrast | 5d_PTX_vs_DMSO_ID6 |
| Database | msigdb_reactome |
| Sort order | pval |
| P value | 2.9e-05 |
| adj. P value | 0.0013 |
| Effect size (AUC) | 0.75 |
| Field | Value |
| Gene set ID | M630 |
| Description | REACTOME_GLUTAMATE_NEUROTRANSMITTER_RELEASE_CYCLE |
| Data set | standard contrasts |
| Contrast | 5d_PTX_vs_DMSO_ID6 |
| Database | msigdb_reactome |
| Sort order | pval |
| P value | 3.4e-05 |
| adj. P value | 0.0014 |
| Effect size (AUC) | 0.69 |
| Field | Value |
| Gene set ID | M10272 |
| Description | REACTOME_IONOTROPIC_ACTIVITY_OF_KAINATE_RECEPTORS |
| Data set | standard contrasts |
| Contrast | 5d_PTX_vs_DMSO_ID6 |
| Database | msigdb_reactome |
| Sort order | pval |
| P value | 0.00038 |
| adj. P value | 0.0099 |
| Effect size (AUC) | 0.83 |
| Field | Value |
| Gene set ID | M838 |
| Description | REACTOME_UNBLOCKING_OF_NMDA_RECEPTORS_GLUTAMATE_BINDING_AND_ACTIVATION |
| Data set | standard contrasts |
| Contrast | 5d_PTX_vs_DMSO_ID6 |
| Database | msigdb_reactome |
| Sort order | pval |
| P value | 6.1e-05 |
| adj. P value | 0.0023 |
| Effect size (AUC) | 0.78 |
| Field | Value |
| Gene set ID | M13015 |
| Description | REACTOME_NOREPINEPHRINE_NEUROTRANSMITTER_RELEASE_CYCLE |
| Data set | standard contrasts |
| Contrast | 5d_PTX_vs_DMSO_ID6 |
| Database | msigdb_reactome |
| Sort order | pval |
| P value | 3.1e-05 |
| adj. P value | 0.0014 |
| Effect size (AUC) | 0.73 |
| Field | Value |
| Gene set ID | M27564 |
| Description | REACTOME_LGI_ADAM_INTERACTIONS |
| Data set | standard contrasts |
| Contrast | 5d_PTX_vs_DMSO_ID6 |
| Database | msigdb_reactome |
| Sort order | pval |
| P value | 0.00015 |
| adj. P value | 0.0046 |
| Effect size (AUC) | 0.78 |
| Field | Value |
| Gene set ID | M27934 |
| Description | REACTOME_ASSEMBLY_AND_CELL_SURFACE_PRESENTATION_OF_NMDA_RECEPTORS |
| Data set | standard contrasts |
| Contrast | 5d_PTX_vs_DMSO_ID6 |
| Database | msigdb_reactome |
| Sort order | pval |
| P value | 4.4e-06 |
| adj. P value | 0.00029 |
| Effect size (AUC) | 0.73 |
| Field | Value |
| Gene set ID | M27944 |
| Description | REACTOME_NEGATIVE_REGULATION_OF_NMDA_RECEPTOR_MEDIATED_NEURONAL_TRANSMISSION |
| Data set | standard contrasts |
| Contrast | 5d_PTX_vs_DMSO_ID6 |
| Database | msigdb_reactome |
| Sort order | pval |
| P value | 0.00039 |
| adj. P value | 0.0099 |
| Effect size (AUC) | 0.73 |
| Field | Value |
| Gene set ID | M27736 |
| Description | REACTOME_SYNAPTIC_ADHESION_LIKE_MOLECULES |
| Data set | standard contrasts |
| Contrast | 5d_PTX_vs_DMSO_ID6 |
| Database | msigdb_reactome |
| Sort order | pval |
| P value | 0.00065 |
| adj. P value | 0.014 |
| Effect size (AUC) | 0.7 |
| Field | Value |
| Gene set ID | M27455 |
| Description | REACTOME_PHASE_0_RAPID_DEPOLARISATION |
| Data set | standard contrasts |
| Contrast | 5d_PTX_vs_DMSO_ID6 |
| Database | msigdb_reactome |
| Sort order | pval |
| P value | 0.0012 |
| adj. P value | 0.021 |
| Effect size (AUC) | 0.68 |

| **Field** | **Value** |
| --- | --- |
| Gene set ID | M5902 |
| Description | HALLMARK_APOPTOSIS |
| Data set | standard contrasts |
| Contrast | 5d_PTX_vs_DMSO_ID6 |
| Database | msigdb_hallmark |
| Sort order | pval |
| P value | 3.2e-11 |
| adj. P value | 4e-10 |
| Effect size (AUC) | 0.63 |
| Field | Value |
| Gene set ID | M24803 |
| Description | GOBP_NEUROTRANSMITTER_LOADING_INTO_SYNAPTIC_VESICLE |
| Data set | standard contrasts |
| Contrast | 5d_PTX_vs_DMSO_ID6 |
| Database | msigdb_go_bp |
| Sort order | pval |
| P value | 0.00054 |
| adj. P value | 0.0065 |
| Effect size (AUC) | 0.93 |
| Field | Value |
| Gene set ID | M29257 |
| Description | GOBP_SPONTANEOUS_NEUROTRANSMITTER_SECRETION |
| Data set | standard contrasts |
| Contrast | 5d_PTX_vs_DMSO_ID6 |
| Database | msigdb_go_bp |
| Sort order | pval |
| P value | 0.00069 |
| adj. P value | 0.0079 |
| Effect size (AUC) | 0.87 |
| Field | Value |
| Gene set ID | M25210 |
| Description | GOBP_NEGATIVE_REGULATION_OF_PERK_MEDIATED_UNFOLDED_PROTEIN_RESPONSE |
| Data set | standard contrasts |
| Contrast | 5d_PTX_vs_DMSO_ID6 |
| Database | msigdb_go_bp |
| Sort order | pval |
| P value | 0.0012 |
| adj. P value | 0.012 |
| Effect size (AUC) | 0.86 |

| **Field** | **Value** |
| --- | --- |
| Gene set ID | M12546 |
| Description | GOBP_ER_NUCLEUS_SIGNALING_PATHWAY |
| Data set | standard contrasts |
| Contrast | 5d_PTX_vs_DMSO_ID6 |
| Database | msigdb_go_bp |
| Sort order | pval |
| P value | 4.1e-11 |
| adj. P value | 3.4e-09 |
| Effect size (AUC) | 0.76 |
| Field | Value |
| Gene set ID | M22492 |
| Description | GOBP_RESPONSE_TO_STEROL_DEPLETION |
| Data set | standard contrasts |
| Contrast | 5d_PTX_vs_DMSO_ID6 |
| Database | msigdb_go_bp |
| Sort order | pval |
| P value | 2.6e-06 |
| adj. P value | 6.8e-05 |
| Effect size (AUC) | 0.81 |
| Field | Value |
| Gene set ID | M25221 |
| Description | GOBP_REGULATION_OF_GLIAL_CELL_MIGRATION |
| Data set | standard contrasts |
| Contrast | 5d_PTX_vs_DMSO_ID6 |
| Database | msigdb_go_bp |
| Sort order | pval |
| P value | 0.00016 |
| adj. P value | 0.0024 |
| Effect size (AUC) | 0.76 |
| Field | Value |
| Gene set ID | M42672 |
| Description | GOBP_REGULATION_OF_GAMMA_AMINOBUTYRIC_ACID_SECRETION |
| Data set | standard contrasts |
| Contrast | 5d_PTX_vs_DMSO_ID6 |
| Database | msigdb_go_bp |
| Sort order | pval |
| P value | 0.00025 |
| adj. P value | 0.0035 |
| Effect size (AUC) | 0.75 |
